# Mechanisms of lung toxicity induced by biomass burning aerosols

**DOI:** 10.1186/s12989-020-0337-x

**Published:** 2020-01-20

**Authors:** Michal Pardo, Chunlin Li, Quanfu He, Smadar Levin-Zaidman, Michael Tsoory, Qingqing Yu, Xinming Wang, Yinon Rudich

**Affiliations:** 1 Department of Earth and Planetary Sciences, Weizmann Institute of Science, 76100 Rehovot, Israel; 2 Electron Microscopy Unit, Weizmann Institute of Science, 76100 Rehovot, Israel; 3 Department of Veterinary Resources, Weizmann Institute of Science, 761001 Rehovot, Israel; 4 State Key Laboratory of Organic Geochemistry and Guangdong Key Laboratory of Environmental Protection and Resources Utilization, Guangzhou Institute of Geochemistry, Chinese Academy of Sciences, Guangzhou, 510640 China; 5 University of Chinese Academy of Sciences, Beijing, 100049 China; 6 Center for Excellence in Regional Atmospheric Environment, Institute of Urban Environment, Chinese Academy of Sciences, Xiamen, 361021 China

**Keywords:** Biomass burning, Wood tar particles, Mitochondria, Oxidative stress, Health effects, Inflammation, Apoptosis, Nrf2

## Abstract

**Background:**

Carbonaceous aerosols emitted from indoor and outdoor biomass burning are major risk factors contributing to the global burden of disease. Wood tar aerosols, namely, tar ball particles, compose a substantial fraction of carbonaceous emissions, especially from biomass smoldering. However, their health-related impacts and toxicity are still not well known. This study investigated the toxicity of the water-soluble fraction of pyrolyzed wood tar aerosols in exposed mice and lung epithelial cells.

**Results:**

Mice exposed to water-soluble wood tar aerosols showed increased inflammatory and oxidative stress responses. Bronchial epithelial cells exposed to the same water-soluble wood tar aerosols showed increased cell death with apoptotic characteristics. Alterations in oxidative status, including changes in reactive oxygen species (ROS) levels and reductions in the expression of antioxidant genes related to the transcription factor Nrf2, were observed and were confirmed by increased levels of MDA, a lipid peroxidation adduct. Damage to mitochondria was observed as an early event responsible for the aforementioned changes.

**Conclusions:**

The toxicity and health effect-related mechanisms of water-soluble wood tar were investigated for the first time in the context of biomass burning. Wood tar particles may account for major responses such as cell death, oxidative stress, supression of protection mechnaisms and mitochondrial damaged cause by expsoure to biomass burning aerosols.

## Background

Atmospheric particulate matter (PM) pollution is one of the leading contributors to the global burden of disease [1–4]. Various sources contribute to the global PM load, including biomass burning [3, 5], which emits large amounts of gases and particulates into the atmosphere. Burning can be natural or human-induced [6]. Natural sources include wildfires, and global warming scenarios project increases in the frequency and intensity of wildfires [7]. These increases may in turn exert feedback and influence global warming [8]. The annual global mortality from vegetation fire smoke is estimated to be approximately 339,000 deaths/year [9]. However, the implications for public health, such as respiratory, cardiovascular, and other morbidity effects, are still unknown [10].

Anthropogenic sources of PM include agricultural operations, industrial processes, and combustion of wood and fossil fuels [3, 5]. During the winter season, wood combustion is a major source of indoor and outdoor PM pollution in many developed and developing countries. Wood stove cooking, which is prevalent in many countries, also increases indoor exposure to biomass burning smoke [11, 12]. Europe and North America are the regions with the highest proportions of outdoor PM that can be traced to residential heating with solid fuels (approximately 21% reported in 2010), and approximately 60,000 premature annual deaths are attributed to ambient air pollution from residential heating with wood and coal in these regions [3, 13].

Wood smoke particles (WSPs) have been studied to evaluate the hazards of exposure to smoke from biomass burning from both wildfires and home wood-burning stoves [4, 12, 14–16]. Existing evidence links emissions from wood and coal burning to severe health effects such as respiratory and cardiovascular mortality and morbidity [17, 18]. Hazardous exposures result from inhalation of gases and particles that are byproducts and intermediates of the combustion process. Analyses of particles emitted from wood combustion have identified inorganic components (sulfates, nitrates, potassium), organic aerosols (OAs) containing various polyaromatic hydrocarbons (PAHs), and other toxic and carcinogenic contaminants [3, 19–21]. Among biomass burning products, amorphous, carbonaceous particles with typical diameters between ten and hundreds of nanometers have been distinguished as a distinct group; these particles are termed “tar balls” [19–21]. Tar balls constitute considerable fraction of biomass burning carbonaceous aerosols in terms of number and mass concentrations [20, 22].

Toxicological evaluations of ambient PM have been extensively conducted, whereas the toxicology and mechanisms of WSPs and related components have been poorly defined. Recent studies on WSP exposure have suggested that WSPs enhance inflammation and oxidative stress responses [4, 23–27]. The oxidative stress paradigm suggests that low levels of ROS/ oxidative stress can induce antioxidant induction to restore redox homeostasis. When this protection is insufficient, the increased stress can induce other mechanisms such as inflammation. For example, macrophages exposed to wood smoke extract generate free radicals and exhibit lipid peroxidation and an inflammatory response accompanied by activation of nuclear factor kappa B (NF-*k*B) and release of tumor necrosis factor (TNF)-α [28]. In addition, oxidative stress has been suggested to be mediated by mitochondria since these organelles are both major intracellular sources of reactive oxygen species (ROS) and ROS targets [29], supporting the paradigm of particle toxicity and oxidative stress.

In the last few years, a major effort has been focused on finding associations between specific components in air pollution and human health effects [25, 27, 30–33]. It was previously suggested that the toxicity of WSPs is strongly dependent on the organic fraction and is associated with organic components other than PAHs [25]. Moreover, water-soluble compounds constitute the primary fraction (up to 80 wt.%) of biomass smoldering smoke particles but have receive less attention than other compounds with regards to their potential toxicological impacts. We hypothesize that the toxicity of biomass burning can be largely attributed to the water-soluble component of wood tar particles.

In this study, we generated wood tar particles from wood pyrolysis and assessed a wide spectrum of toxicity endpoints (overall toxicity and markers for inflammation, oxidative stress and mitochondrial function) in vivo (acute response) in exposed mice and in vitro in human BEAS2B lung epithelial cells. To the best of our knowledge, the toxicity of wood tar in general and of the water-soluble component in particular has not been previously reported.

## Results

### Water-soluble wood tar extract characterization and exposure assessment

Wood tar materials were generated by pyrolysis of wood under conditions that simulated the smoldering process. The water-soluble fraction of the wood tar material was extracted to generate an atmospherically relevant wood tar solution. The solution was atomized by a TSI atomizer and then dried to generate a flow of dry particles (Additional file 1: Figure S1). The chemical composition of the particles generated from the extract was qualitatively and quantitatively characterized, as presented in the supporting information (Additional file 1: Figure S1-S2 and Table S1).

Mice (in particular, their heads and noses) were exposed to wood tar aerosols in an individual exposure system (See Additional file 1 for details). The mice were exposed to each concentration of nebulized wood tar aerosols (2 mg/ml or 10 mg/ ml) for 15 min. The methods for wood tar aerosol exposure assessment and quantification are shown in Additional file 1: Figure S3. The inhaled doses were assessed by monitoring the size distributions of the wood tar aerosols using a scanning mobility particle sizer (SMPS, TSI, MN, USA). Size distribution measurement showed that the particle mass mode diameters ranged from 300 to 400 nm with a mobility mode diameter of ~ 200 nm. These particle sizes are similar to those of typical biomass burning and urban environmental pollution aerosols [34, 35]. The calculated inhaled dose for a single exposure for each mouse was 16 μg for the 2 mg/ml initial concentration solution and approximately 677 μg for the 10 mg/ml initial concentration solution (SI).

### Inflammatory responses following exposure to wood tar aerosols

**Fig. 1:**
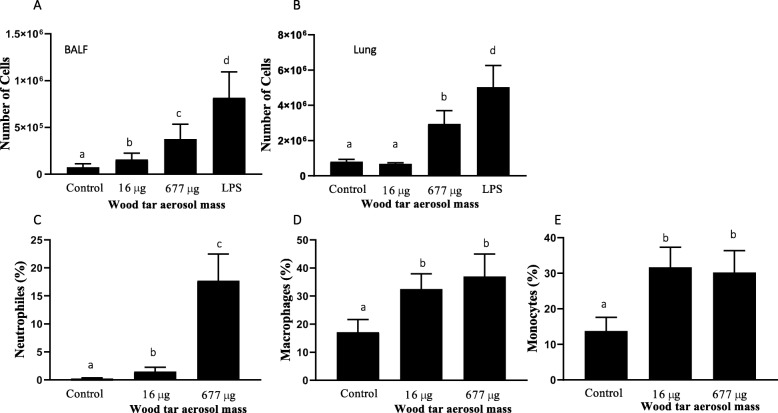
Inflammatory responses in mice following exposure to aerosols generated from water-soluble wood tar extract. Mice were exposed to wood tar solution-generated particles using an individual single exposure model. For each exposure, the initial concentration of the water-soluble extract from wood tar was 2 mg/ml or 10 mg/ml. Aerosols were generated via nebulization of these solutions and directed to six mice for each of the concentrations tested (*n* = 6). Lipopolysaccharide (LPS) was used as a positive control (*n* = 4). PBS was used as the negative control. **a** Total cell number in BALF and **b** total cell number in lung tissue. Further verification of the different populations was performed by flow cytometry of the collected cells stained with different markers. **c** Neutrophil percentage. **d** Macrophage percentage. **e** Monocyte percentage. The data are expressed as the mean ± SEM. Means marked with different letters are significantly different from each other at *p* < 0.05

Mice exposed to wood tar aerosols showed increased inflammatory responses, as indicated by increased total cell counts in both bronchoalveolar lavage fluid (BALF) and lung tissue and increased neutrophil, macrophage, and monocyte levels (Fig. 1 and Additional file 1: Figure S4). To confirm the increase in the inflammatory response observed in BALF, the gene expression of interleukin (IL)-1β, TNF-α, and IL-6, which are inflammatory cytokines involved in PM-induced inflammation, was investigated by real-time PCR [15, 16, 31]. Exposure to aerosols generated from the high-concentration wood tar solution (677 μg dose) increased the transcript levels of IL-1β, TNF-α, and IL-6 (Table 1 and Additional file 1: Figure S5) in lung tissue, whereas exposure to aerosols generated from the low-concentration solution (16 μg dose) increased only the transcription levels of IL-1β.

**Table 1:**
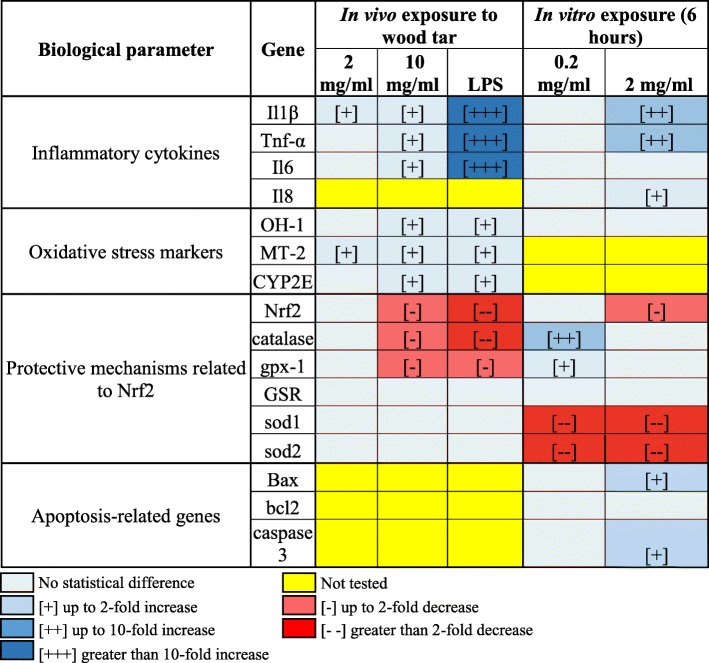
Summary table for biological responses in vivo and in vitro

^*^Pluses and minuses are statistical significance compared to their control

In addition to the in vivo experiments, in vitro experiments were performed in which human lung epithelial cells (BEAS2B) were exposed to the 2 mg/ml water-soluble extract of wood tar solution that increased inflammatory gene expression (IL-1β, TNF-α, and IL-8) (Table 1 and Additional file 1: Figure S6)*.*

### Water-soluble wood tar induced cell death in lung epithelial cells

**Fig. 2:**
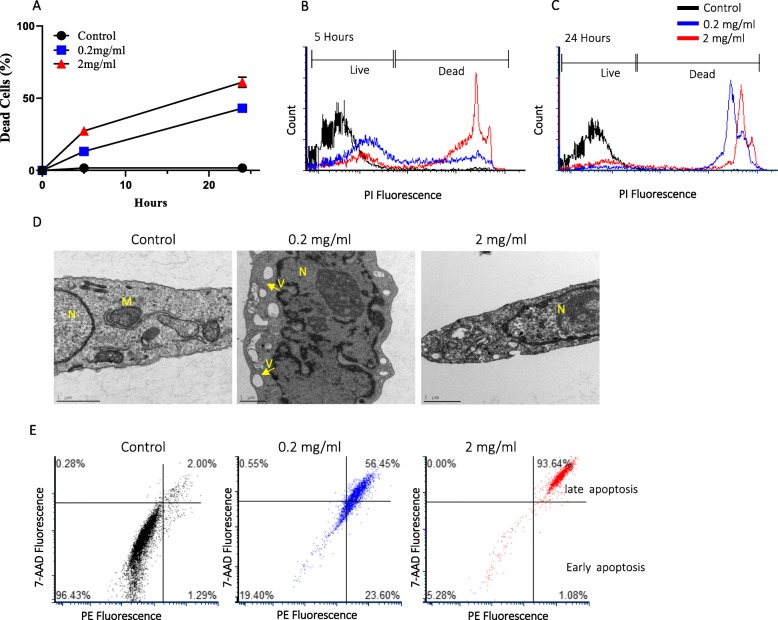
Cell toxicity after exposure to wood tar extract. Cells were exposed to water-soluble extracts of a wood tar solution with a concentration of 0.2 mg/ml or 2 mg/ml for 5 or 24 h prior to the analysis, as specified. **a** PI-positive cells were detected by flow cytometry (ZE5 Cell Analyzer, Bio-Rad) as a measurement of cell viability. **b** Flow cytometry histogram after 5 h of exposure. **c** Flow cytometry histogram after 24 h of exposure. The data are expressed as the mean ± SD. Means marked with different letters are significantly different from each other at *p* < 0.05. **d** TEM images of control (blank-treated) cells, 0.2 mg/ml wood tar extract-treated cells, and 2 mg/ml wood tar extract-treated cells after 5 h of exposure. M, mitochondria; N, nucleus; V, vacuoles. **e** Flow cytometry histogram of apoptosis stages determined after 5 h of exposure using the Guava Nexin Reagent

Five and 24 h after exposure, approximately 15 and 45% of the cells stained positive for propidium iodide (PI), respectively, and were considered dead (Fig. 2a) (for the 0.2 mg/ml concentration). A significant decrease of 55% in cell viability was observed following 24 h of exposure to the 2 mg/ml water-soluble wood tar solution (Fig. 2a-c.). Similarly, a WST-1 assay showed reduced survival after exposure to the wood tar extract (at the same concentration), albeit to a lesser extent, possibly because the PI dye was more sensitive than the WST-1 dye (Additional file 1: Figure S7).

Transmission electron microscopy (TEM) of BEAS2B cells exposed to different concentrations (0.2 mg/ml and 2 mg/ml) of water-soluble wood tar extract solution at different time points (i.e., 5 and 24 h) showed clear changes in the organelles of the exposed cells compared to their controls (Fig. 2d). Prominent changes were observed in the mitochondria. The shapes of the mitochondria in the control cells were heterogeneous, with numerous visible cristae, and the inner and outer mitochondrial membranes appeared intact. Exposure to 0.2 mg/ml wood tar extract for 5 h induced marked mitochondrial abnormalities, such as swelling with disarrangement and distortion of cristae. Strikingly, multiple vesicles appeared in the cytosol, and this effect was observed to a greater extent after 24 h than after 5 h (Additional file 1: Figure S7). Some of these vesicles could have been remnants of damaged mitochondria. In addition, the nuclei were visible, with evident chromatin condensation. Nevertheless, the outer membranes were not disrupted. All these observations may indicate that the cells were undergoing cell death through apoptosis [36]. Exposure to 2 mg/ml wood tar extract for 5 h also induced profound changes in cell structure and permeabilization of the plasma membrane. There were progressive discontinuities that could cause swelling of the cells as well as organelle disruption. Nevertheless, the outer membranes remained intact, suggesting an acute response of cell death (Fig. 2d).

To further characterize the cell death mechanism, cells were stained with annexin V and 7-aminoactinomycin D (7-AAD) to distinguish between the different apoptosis stages (early and late). The proportion of cells in late apoptosis was highest among the wood tar-treated cells, particularly those treated with the 2 mg/ml concentration (Fig. 2e). It was found that 0.2 mg/ml wood tar induced both early and late apoptotic cell death. Apoptosis was also confirmed by the expression of the Bcl-2-associated X protein (BAX; a proapoptotic factor) and caspase-3 genes, which increased after exposure to 2 mg/ml wood tar extract (Table 1 and Additional file 1: Figure S6).

### ROS alterations and oxidative stress after exposure to water-soluble wood tar

**Fig. 3:**
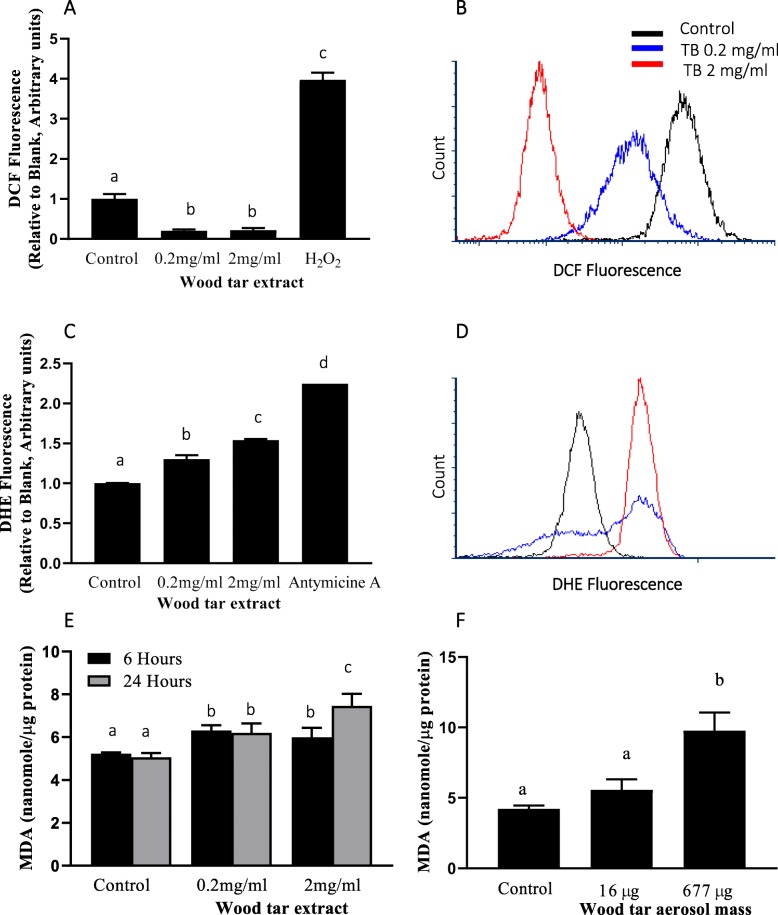
Oxidative stress after exposure to wood tar extracts. **a** Intracellular ROS were measured using H_2_DCF-DA, and detection was performed by flow cytometry (ZE5 Cell Analyzer, Bio-Rad). **b** Flow cytometry histogram for DCF fluorescence. **c** Superoxide anions were measured using DHE, and detection was performed by flow cytometry (ZE5 Cell Analyzer, Bio-Rad). **d** Flow cytometry histogram for DHE fluorescence. Lipid oxidation was measured in **e** cells exposed to wood tar suspension and **f** mice exposed to wood tar solution as described in the methods section. The data are expressed as the mean ± SD. Means marked with different letters are significantly different from each other at *p* < 0.05

We investigated the potency of the wood tar extract in inducing ROS formation in exposed BEAS2B cells using different probes. Cellular ROS production was evaluated through measurement of dihydrodichlorofluorescein (DCFH) oxidation after 5 h of exposure. The exposed cells exhibited reduced hydrogen peroxide production capacity (usually considered an indicator of total ROS) (Fig. 3a-b); the hydrogen peroxide levels in the exposed cells were 2-fold lower than those in the control cells. In addition, dihydroethidium (DHE) oxidation was assessed to detect superoxide anions. In contrast to hydrogen peroxide production, superoxide anion production was increased by the wood tar extracts. This finding may suggest impaired dismutation of superoxide to hydrogen peroxide (Fig. 3c-d).

To further explore the changes in oxidative stress status in vitro and in vivo, real-time PCR analyses for genes that are considered to be markers of oxidative stress and genes that are related to the Nrf2 protection mechanism were performed (Table 1, Additional file 1: Figure S5 and Figure S6). Exposure of BEAS2B cells to the wood tar extracts did not increase any oxidative stress markers but elicited different responses in the expression of genes related to Nrf2. Exposure to the low-concentration (0.2 mg/ml) wood tar extract increased the expression of Nrf2-related genes such as catalase and glutathione peroxidase-1 (GPx-1), whereas exposure to the high-concentration (2 mg/ml) wood tar extract reduced the expression levels of Nrf2 and catalase. Increases in oxidative stress markers such as heme oxygenase-1 (HO-1), Metallothionein-2 (MT-2) and Cytochrome P450 2E (CYP2E) were observed in mice exposed to water-soluble wood tar aerosols. Genes related to Nrf2 signaling (Nrf2, catalase, and GPx-1) showed reduced levels in mice exposed to wood tar aerosols (Table 1, Additional file 1: Figure S5).

To assess whether the cells were subjected to oxidative stress, the levels of MDA, a lipid peroxidation adduct, were examined after exposure to the wood tar extracts. A minor but significant increase in MDA levels was observed after exposure to the 0.2 mg/ml wood tar extract for both 5 and 24 h. A higher increase in MDA levels was observed after 24 h of exposure to the 2 mg/ml wood tar extract (Fig. 3e). In addition, exposure of mice to 16 and 677 μg of aerosols from the water-soluble wood tar extract solutions increased lung lipid peroxidation levels in a dose-dependent manner (Fig. 3f).

### Water-soluble wood tar damages mitochondria

**Fig. 4:**
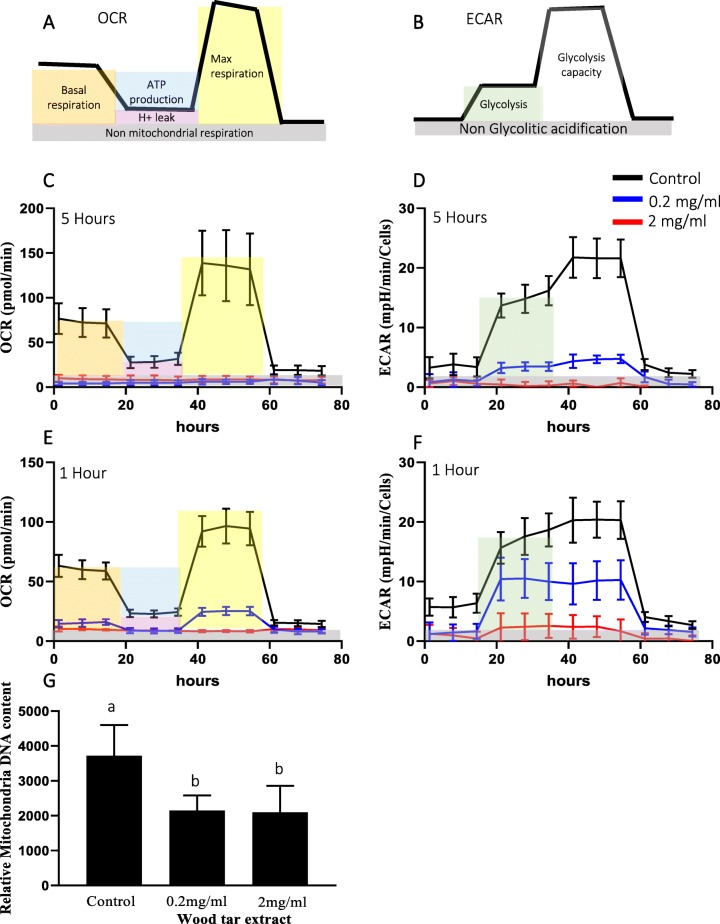
Mitochondrial response following exposure to wood tar extracts. Cells were exposed to water-soluble extracts from wood tar solution (at a concentration of 0.2 mg/ml or 2 mg/ml) for one or 5 h prior to analysis, as specified. Determination of the mitochondrial OCR (a measure of mitochondrial respiration) and ECAR (a measure of glycolysis) was performed with a Seahorse analyzer. **a** Description of the mitochondrial respiration (mitochondrial stress) and **b** Seahorse glycolysis assays. Selected results showing **c** the basal and mean OCR and **d** the basal and mean ECAR following injection of inhibitors and substances after 5 h of exposure are shown. **e** The OCRs after 1 h of exposure and **f** the ECARs after 1 h of exposure are shown. **g** MtDNAcn. The data represent the mean ± SD. These experiments were performed in triplicate and repeated twice

Mitochondrial functions were evaluated in BEAS2B cells following exposure to wood tar extracts using a Seahorse analyzer (Fig. 4). As shown in Fig. 4 and the Additional file 1: Figure S8, 5 h of exposure of BEAS2B cells to wood tar extracts resulted in complete inhibition of the oxygen consumption rate (OCR, red and blue lines) for both concentrations tested (Fig. 4c). This observation further supports the notion that wood tar extracts induce toxicity via mitochondria-related mechanisms.

Cellular metabolism is an important determinant of cell survival and function and comprises oxidative phosphorylation and glycolysis, which are the two main sources of ATP in cells [37]. We therefore measured the glycolysis rate (as the extracellular acidification rate, ECAR) after exposure to wood tar extracts (Fig. 4). ECAR, which reflects glycolysis, was significantly reduced after 5 h of exposure, indicating that water-soluble wood tar impairs cellular bioenergetics (Fig. 4d). Cellular bioenergetics measurements were also performed after 1 h of exposure to wood tar extracts and revealed significant decreases in both the OCR and the ECAR (Fig. 4e-f).

To further study the influence of wood tar particles on mitochondria, mitochondrial DNA copy numbers (MtDNAcns) were evaluated. Exposure to the wood tar extracts reduced the MtDNAcns by approximately 50% in BEAS2B cells for both concentrations measured (Fig. 4g).

## Discussion

Wood tar aerosols are typical and abundant particles in biomass burning emissions. In our study, we generated tar aerosols that served as proxies for particles from smoldering carbonaceous materials. The goal of this study was to investigate the acute cytotoxic effects of the water-soluble fraction of a smoldering wood tar solution both in vivo and in vitro. To achieve this goal, we characterized the physical and chemical properties of the generated wood tar extract [19] and exposed mice and lung epithelial cells to this extract.

### Wood tar exposure chemical assessment

Based on our previous and current studies, wood tar aerosols generated by nebulizing the water-soluble extract from pyrolyzed wood are good proxies for atmospheric wood tar particles [19, 20, 38–40]. In this study, the water-soluble wood tar extracts were used in two different concentrations for the in vivo experiments. The calculated inhaled doses (for each mouse) were 16 μg and 677 μg of dry wood tar particles for the low- and high-concentration extract solutions, respectively. The concentrations used were randomly selected since exposure to wood-burning activity can vary substantially between different households, from day to day or during exposure to wildfire emissions. Consequently, it is difficult to simulate exposures that are relevant on time scales of hours or days. It has previously been suggested that the concentration of tar balls from biomass plumes is approximately 80% of that of the smoke particles emitted from smoldering biomass close to the source [20], and the concentration decreases with distance and dilution. Additionally, tar ball aggregates compose a significant fraction (27%) of samples collected in a plume of the Whitewater-Baldy Complex fire in New Mexico [22]. Therefore, exposure of mice to our conditions for 15 min is an acute exposure equivalent to days or months of exposure to real biomass burning air pollution according to actual measurements in domestic and field environments [38, 41].

In addition, long term exposure to biomass burning smoke was previously associated with chronic obstructive pulmonary disease (COPD). However, the effects of acute exposures have not been thoroughly studied. Acute exposures as performed in this study suggest possible increased susceptibility to lung disease.

### Inflammatory responses after exposure to wood tar aerosols

Previous studies have indicated that exposure to WSPs in indoor air and from wildfires, burning of biomass and air pollution may impact health [1, 2, 4, 14, 15, 27, 32, 42]. It has been shown that WSPs are associated with systemic and pulmonary inflammation [4, 14, 42], as healthy subjects who are exposed develop increased levels of neutrophils in BALF and blood [42]. The results of this study also showed an increased inflammatory response, with increased neutrophil, macrophage and monocyte counts, in exposed mice (Fig. 1).

In addition, an increase in inflammatory gene expression was observed in cells exposed to wood tar extract (Table 1 and Additional file 1: Figure S6). Some in vitro studies on exposure to water-soluble extracts from collected biomass burning particles have also indicated that exposure induces onset of an inflammatory response similar to that observed in our study [4, 28, 43, 44], while other studies have shown only a minor inflammatory response following exposure to biomass burning extracts [42, 45, 46]. The combined results from this study suggested that the inflammatory response was increased in both the in vivo and in vitro exposures. However, the inflammatory response appeared milder in the cultured cells than in the mice in vivo expsoure (Table 1 and Additional file 1: Figure S5), suggesting that exposure to wood tar induces a systemic response that is amplified in vivo during exposure.

### Cytotoxicity of wood tar to lung epithelial cells

In vitro studies have demonstrated that biological effects induced by water-soluble extracts from biomass burning particles can generate ROS [46] and induce DNA damage [15, 23, 25, 46]. In our study, the acute cytotoxicity of the wood tar extracts (at concentrations of 0.2 and 2 mg/ml) was investigated by using the permeable dye PI, a WST-1 assay and electron microscopy with BEAS2B cells. Exposure to wood tar extracts resulted in cell death in a time- and dose-dependent manner (Fig. 2). These results are supported by a previously published study that showed a decrease in mouse macrophage viability (through PI staining) after exposure to PM1 samples from wood log combustion [15].

Apoptotic cell death is characterized by biochemical events leading to the formation of apoptotic bodies. These bodies are removed by phagocytic cells. Unless these bodies are removed, the cell contents will contact the surrounding cells and damage them by releasing potentially inflammatory intracellular components [47, 48]. Failure of apoptotic cell clearance can lead to late apoptosis (also referred to as secondary necrosis) [47, 48]. Dying cells can be characterized as being in early apoptosis, in which phosphatidylserine is expressed on the cell surface and the plasma membrane remains intact. Early apoptotic cells can become late apoptotic cells if the plasma membrane becomes permeabilized [47, 48]. Our results showed that cells exhibited apoptotic characteristics following exposure to the wood tar extract (Fig. 2). The importance of apoptosis in PM toxicity has been previously reported [49, 50]. Our results suggest that apoptosis occurs after exposure to water-soluble wood tar extracts and may result in oxidative damage. As these results indicate a late apoptosis response, clearance of apoptotic cells is likely delayed or impaired. Our findings are also consistent with those of a previous report [51] indicating that exposure of animals to WSPs increased apoptosis in BALF macrophages and lung tissue.

### ROS formation, oxidative stress and mitochondrial damage following exposure to water-soluble wood tar

A central paradigm of particle toxicology, particularly PM pollution toxicology, is formation of ROS that leads to inflammation and other adverse health effects [30, 52]. ROS include the superoxide anion, hydrogen peroxide, and hydroxyl radicals, all of which can react and oxidize different biological targets [29]. In this study, we observed increased superoxide anion concentrations and decreased hydrogen peroxide concentrations after exposure. The decreased oxidation of the DCFH fluorophore following exposure to wood tar extracts could be related to the activity of one or two antioxidant enzymes that prevent accumulation of superoxide in the cytosol (superoxide dismutase [SOD1], Cu/ZnSOD) and in the mitochondria (SOD2, MnSOD). Indeed, decrease in both SOD1 and SOD2 gene expression were observed following exposure to wood tar extract.

Increases in the levels of different ROS species may lead to oxidative stress through disruption of the balance between oxidant and antioxidant molecules, leading to tissue damage (to DNA, lipids and proteins [31, 32, 53](. It has previously been shown that the transcription factor Nrf2 and its related genes are involved in the response to PM exposure [31, 32]. We have suggested that repeated exposures to PM may exhaust the Nrf2 antioxidant defense system, thus leading to oxidative stress. Therefore, the expression of antioxidant genes related to Nrf2 was analyzed by real-time PCR after exposure to wood tar extracts (Table 1, Additional file 1: Figure S5 and Figure S6). Exposure of BEAS2B cells to the highest concentration (2 mg/ml) of wood tar extracts reduced Nrf2-related gene expression. Wood tar extract at the 0.2 mg/ml concentration did not reduce Nrf2-related gene expression; on the contrary, it increased the expression of catalase and GPX genes. This finding may imply the induction of protection mechanisms. In addition to the cells, the mice exposed to wood tar aerosols also exhibited reduced levels of phase II protective genes (Table 1, Additional file 1: Figure S5). Taken together, the reductions in SOD enzymes and decreases in phase II protection enzymes related to the Nrf2 pathway following exposure to wood tar extracts may support the notion that the exposure reduced cellular antioxidant capacity.

Accumulation of superoxide in cells is associated with oxidative stress [29]. The cells in this study were subjected to oxidative stress after exposure to wood tar extracts that led to actual oxidative damage (increased MDA levels), particularly the cells exposed to the higher concentration (2 mg/ml). Increased MDA levels was also observed in mice exposed to wood tar. Several other studies have also shown increases in MDA levels after exposure to WSPs; for example, WSPs released during cooking with fuelwood have been found to increase plasma MDA levels in women from northeast India [12]. In addition, following exposure to wood smoke, alveolar MDA levels increase in human subjects [24]. Another study showed that wood smoke generated hydroxyl radicals (OH^·^) and induced MDA formation (lipid peroxidation) [28]. In this study, mild changes in lipid peroxidation were observed and elevated levels of superoxide anion were measured within cells after exposure to the extract. As hydroxyl radicals are thought to be the main radicals responsible for damage, this finding may explain the low levels of lipid peroxidation observed in this study.

Mitochondria are the major cellular sources of ROS, which are generated as byproducts during normal respiration [26, 29, 54]. The alterations in ROS levels, the structural changes in the mitochondria observed using TEM and even the reductions in SOD2 levels may suggest that exposure to water-soluble wood tar can lead to mitochondrial damage. Damage to mitochondria could have been the result or even the cause of the changes in ROS. The mitochondrial damage observed using the Seahorse analyzer following 1 h of exposure may suggest that mitochondria are organelles that respond early to exposure. This finding may imply that increased ROS levels, alterations in oxidative stress status, and cell death via apoptosis are consecutive events following mitochondrial damage. We have previously shown that exposure of cells to organic extracts containing high PAH levels reduces the rates of cellular bioenergetic processes (both the OCR and the ECAR) [33]. The water-soluble wood tar extracts used in this study had low PAH contents. However, they were more toxic with respect to cellular bioenergetics than the organic extracts from samples from Beijing used in our previous study, which were collected in winter and dominated by PAHs from coal combustion [33].

MtDNAcn can be used as another indicator of mitochondrial damage because it correlates with the size and number of mitochondria in a cell and may change under different cellular energy demands or different physiological or environmental conditions [33, 54]. Recent studies have correlated ambient PM exposure with mitochondrial DNA damage [26, 33, 54]. Similar to our study, a previous study found that personal exposure to fine PM and benzo [*a*] pyrene from indoor air pollution reduced MtDNAcns in leukocytes of women from China [55].

## Conclusions

**Fig. 5:**
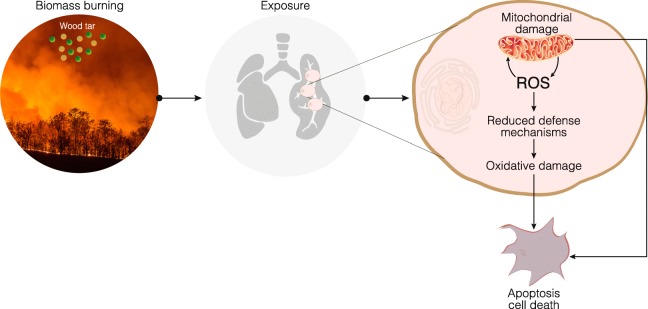
Illustration of water-soluble wood tar extract toxicity. Wood tar generated from biomass burning increases inflammation in lung tissue and lung epithelial cells. Following exposure, mitochondria are damaged, and increases in ROS and decreases in defense mechanisms lead to oxidative damage and cell death

Biomass burning affects air quality. As a result, outdoor and indoor exposure to biomass burning smoke is an important and growing health risk factor. As previously suggested, the responses induced by the organic fraction of biomass burning smoke are not linked solely to PAH content; rather, they are also associated with other organic compounds [25, 56]. High-resolution aerosol mass spectrometry (HR-AMS), TEM, and Fourier transform infrared spectroscopy (FTIR) results have suggested that wood tar aerosols may serve as proxies for biomass burning aerosols [19]. As found in this study, wood tar aerosols can account for the major responses observed in many studies following exposure to biomass burning particles [14, 15, 25]. Considering all these data, we propose a mechanism of action in which wood tar emissions generated by biomass burning exert toxic effects both in vivo in mice lungs and in vitro in lung cells. Our results suggest that mitochondria play key roles in the early response to wood tar exposure, as mitochondrial function is dramatically reduced immediately following exposure. Damage to mitochondria is also evidenced by reduced MtDNAcns. Consequently, extracts from pyrolyzed wood tar induce oxidative stress and result in cell death by apoptosis in a dose- and time-dependent manner. Cellular homeostasis is also interrupted by reductions in the levels of Nrf2-related protective genes. If cells cannot manage the high concentrations of wood tar and implement protective mechanisms, cell death eventually occurs (Fig. 5).

Notably, the results of the in vivo mouse and in vitro cell exposures in this study may underscore the severity of the health impacts of wood tar extracts, especially considering that the less-polar fraction with greater enrichment of the more-toxic PAHs and their derivatives was not included in our investigation. However, the water-soluble fraction may be more relevant to health, as the particles can be efficiently deposited and dissolved in the respiratory system.

## Methods

### Wood tar generation and characterization

Wood tar was generated and characterized as previously described [19, 39, 40]. Briefly, wood pellets (Hallingdal Trepellets; water content 6.55 wt.%; length 2~3 cm, diameter 0.2–0.3 cm) were pyrolyzed at 550 °C, and the dry distilled tar materials were collected using a water-cooled trap. The water-soluble fraction of the tar materials was extracted with MilliQ water (18 MΩ, sterilized by 185 nm UVA irradiation) and filtered using 0.45 μm and 0.2 μm syringe filters in sequence (polytetrafluoroethylene [PTFE] membrane, Pall Corporation) to remove impurities and PM. Afterward, the filtered solutions were further centrifuged to remove any suspended colloidal particles (2500 rps for 4 min at − 2 °C). Finally, the extracted tar solution was freeze-dried to obtain the water-soluble tar material in a semisolid form. The water-soluble extracts were redissolved and diluted to a 20 mg/L stock solution using sterilized ultrapure water. Before testing, the chemical compositions of the processed wood tar extracts were extensively characterized using FTIR (Thermo Scientific Nicolet 6700) and multiple mass spectrometry applications, e.g., high-resolution time of flight aerosol mass spectrometry (HR-ToF-AMS, Aerodyne) and GC-MS. The detailed measurements and results are given in the Additional file 1: Figure S1 and S2.

### In vivo exposure to wood tar aerosols

The study was approved by the Institutional Animal Care and Use Committee (IACUC) at the Weizmann Institute of Science. Seven-week-old female C57BL/6 mice were purchased from Harlan Laboratories (Rehovot, Israel). One week after arrival, the mice were exposed to the water-soluble tar aerosol. The exposure system used in this study was made of Plexiglas and was divided into six sections for six individual animals. The main inlet was connected to a nebulizer at its top, and the nebulized particles were uniformly distributed throughout the chambers. The outlet of the chamber was connected to a vacuum trap (Additional file 1: Figure S3a). Water-soluble tar extract or phosphate buffer solution (PBS) was aerosolized into the chamber, and the mice were allowed to breathe air containing the aerosol without restraint or anesthesia. PBS Aerosol containing *Escherichia coli* lipopolysaccharide (LPS) (0.5 mg/mL; L2630, Sigma) was used as positive control. Complete aerosolization of the solution was achieved in 15 min. The aerosol generation and exposure system was designed to ensure exposure to the head and nose only, with minimal effect on the skin or fur. The mice were exposed once to the tar aerosol using a solution with one of two different initial concentrations: 2 mg/ml or 10 mg/ml. The details of the exposure assessment, particle size distribution and mass concentrations are described in the Additional file 1: Figure S3. After completion of the respiratory exposure, the mice were returned to their cages.

Twenty-four hours following the exposure, the mice were sacrificed with an overdose of ketamine/xylazine (20 mg/kg and 10 mg/kg body weight, respectively), and whole-body perfusion with PBS was performed. BALF was extracted as previously described [32]. Briefly, the lungs and tracheas were exposed by dissection, and a tracheal cannula was inserted. The lungs were lavaged with PBS, and the cells were separated by centrifugation. The cells were resuspended in 100 μl of sterile saline. The lungs and liver were extracted.

### Lung homogenate preparation and flow cytometry (FACS) analysis

Mouse lungs were removed and washed in RPMI medium containing 1 mg/ml collagenase type 4, 0.75 mg/ml hyaluronidase (Sigma), and 0.02 mg/ml DNase I (Roche). Then, the lungs were minced, incubated at 37 °C for 45 min and then filtered through a 100 μm cell strainer. Lung cells and BALF cells were suspended in red blood cell lysis buffer and then washed twice with FACS buffer. Conjugated anti-mouse antibodies (CD45-PerpCP, CD11b-PE, F4/80-APC/Cy7, PE/Cy7-CD115 and Ly6G-APC) (BioLegend, San Diego, CA) were used. The samples were washed and analyzed with a ZE5 Cell Analyzer (Bio-Rad). Approximately 10^4^ cells were collected from each sample. To identify the different populations, the cells were gated for CD45 and CD11b membrane staining and then gated with for F4/80, CD115 and Ly6G staining (for macrophages, monocytes and neutrophils, respectively). The presented population in percentage is calculated for Macrophages as %Gated, from Cd45+ & F4/80, for Monocytes as %Gated, Cd45+ & CD115, and for Neutrophils as %Gated, Cd45+ & CD11b high & Ly6G high.

### RNA extraction and real-time PCR

Total RNA was extracted from the lungs using TRI reagent according to the manufacturer’s recommendation. Total RNA (1 μg) was reverse-transcribed into cDNA using random hexamers (Applied Biosystems, CA, USA). The cDNA samples were amplified using SYBR Green qPCR Mix (Applied Biosystems, CA, USA) in a StepOnePlus real-time PCR system (Applied Biosystems, CA, USA). The relative expression was normalized using the expression levels of β-actin and HPRT. The PCR data was analyzed using StepOnePlus real-time PCR software V2.3 (Applied Biosystems, CA, USA). The primer sequences are listed in the Additional file 1: Table S2.

### Oxidative damage

Oxidative damage in lung tissue and cell cultures was evaluated by examining lipid peroxidation using the thiobarbituric acid (TBA) method, as previously described [31]. Absorbance was measured in a microplate reader (Bio-Tech Instruments, VT, USA) at 532 nm. A standard curve was created with MDA tetrabutylammonium salt (Sigma-Aldrich, MO, USA).

### Cell culture and exposure

The human lung bronchial cell line BEAS2B (ATCC® CRL-9609™) was grown in DMEM (Gibco, Thermo Fisher Scientific, MA, USA) supplemented with 10% fetal bovine serum (FBS) and 5 μg/ml penicillin/streptomycin (Biological Industries) at 37 °C in a humidified atmosphere consisting of 95% air and 5% CO_2_.

BEAS2B cells were exposed to wood tar suspension in serum-free medium with salts/glucose; the medium comprised 50 mM HEPES, 100 mM NaCl, 5 mM KCl, 2 mM CaCl_2_, and 5 mM glucose (pH 7.2 prior to use to maintain osmolarity). The cells were exposed to wood tar suspensions at 0.2 mg/ml and 2 mg/ml concentrations and to blank extracts, which underwent the same procedures as the suspensions but with water and were used as controls. The working concentration were determined in preliminary tests to set suitable range limits. Cell death was measured after exposure for both 5 and 24 h. However, since a substantial number of cells died within 24 h, all the other assays were performed after 5 h exposure.

### Determination of cell viability and cell death mechanisms

The DNA-intercalating dye PI, which is excluded by viable cells, was used. Flow cytometry analysis (ZE5 Cell Analyzer, Bio-Rad) was used to evaluate cell viability with the following fluorescence settings: excitation (Ex) at 488 nm and emission (Em) at 610 nm [57]. The data were collected from 10,000 cells.

In addition, a WST-1 assay was used according to the manufacturer’s instructions (Abcam, Cambridge, UK.) Absorbance was measured in a microplate reader (Bio-Tech Instruments, VT, USA) at 440 nm and 650 nm.

To evaluate the type of cell death, Annexin V (V-PE) and the impermeant dye 7-AAD (Guava Nexin Reagent, Guava Technologies) were used to distinguish between the early/late apoptosis stages and cell death mechanisms. Fluorescence was measured at an Ex of 488 nm and an Em of 575 nm. The data were collected from 10,000 cells.

### TEM analysis

Cells were fixed with 3% paraformaldehyde and 2% glutaraldehyde in 0.1 M cacodylate buffer containing 5 mM CaCl_2_ (pH 7.4) and then post fixed in 1% osmium tetroxide supplemented with 0.5% potassium hexacyanoferrate trihydrate and potassium dichromate in 0.1 M cacodylate for 1 h. The cells were then stained with 2% uranyl acetate in water for 1 h, dehydrated in graded ethanol solutions and embedded in Agar 100 epoxy resin (Agar Scientific Ltd., Stansted, UK). Ultrathin sections (70–90 nm) were viewed and photographed with an FEI Tecnai SPIRIT (FEI, Eidhoven, Netherlands) transmission electron microscope operated at 120 kV and equipped with an EAGLE charge-coupled device (CCD) camera.

### Measurement of intracellular ROS

Following 5 h of exposure to wood tar suspension, intracellular ROS were detected. For detection with dichlorodihydrofluorescein diacetate (H_2_DCF-DA), the cells were incubated with 25 μM H_2_DCF-DA for 30 min at 37 °C. The dichlorofluorescein (DCF) fluorescence was recorded using flow cytometry at an Ex/Em of 488/532 nm [57]. Hydrogen peroxide (H_2_O_2_) was used as a positive control. For detection with DHE, the cells were incubated with 25 μM DHE for 30 min at 37 °C [58]. DHE emission was recorded using flow cytometry with an Ex/Em of 488/575 nm [59, 60]. Antimycin A (AA) was used as a positive control. The data were collected from 10,000 cells.

H_2_DCF is more specific for hydrogen peroxide than to other ROS since its oxidation depends on intracellular peroxidase activity; thus, H_2_DCF has high reactivity with hydrogen peroxide, lipid hydroperoxide, and hydroxyl radicals and low reactivity with superoxide anions [58, 61]. DHE is a redox-sensitive probe that has been widely used to detect intracellular superoxide anions. The superoxide anion (O·̄_2_) reacts with DHE to form an oxidized product and leads to the enhancement of fluorescence [59, 60].

### Mitochondrial physiology

Mitochondrial bioenergetics and function were measured using an XF96 Extracellular Flux Analyzer (Seahorse Bioscience, North Billerica, MA, USA) according to the manufacturer’s instructions, as previously described [33]. BEAS2B cells were seeded for 24 h in specific Seahorse tissue culture plates at a density of 6 × 10^4^ cells/well. Following exposure to wood tar suspension for 5 h, both mitochondrial respiration (measured as the OCR) and glycolysis (measured as the ECAR) were evaluated. The OCR was evaluated after adding 0.5 μM oligomycin, 1 μM carbonyl cyanide 4-(trifluoromethoxy) phenylhydrazone (FCCP), and 0.5 μM rotenone. The ECAR was evaluated after adding 10 μM glucose, 1 μM oligomycin and 50 mM 2-Deoxy-D-glucose. Both the OCR and the ECAR were normalized to the number of cells per well using Cyquant staining (Thermo Fisher Scientific, Waltham, MA, USA).

### MtDNAcn

DNA extraction was performed with a DNeasy Blood and Tissue Kit (Qiagen). Real-time PCR for human tRNA Leu (UUR) and β2-microglobulin, representing mitochondrial and nuclear DNA genes, respectively, was performed as previously described by [62]. The primers used are listed in Additional file 1: Table S1.

### Statistical analysis

The mouse results are expressed as the mean ± standard error of the mean (SEM). The cell culture results are expressed as the mean ± standard deviation (SD) of at least three experiments. Differences between two group means were tested by Student’s *t*-test, and one-way ANOVA was used for multivariable analyses. Differences were considered significant at a probability level of *p* < 0.05 using Tukey's honestly significant difference (HSD) test. The statistical analyses were performed and the graphs were generated in GraphPad#8 software (GraphPad Software, La Jolla, CA, USA).

## Supplementary information

**Additional file 1.** **Figure S1.** HR-Tof-AMS spectra for water-soluble wood tar aerosol. **Figure S2.** FT-IR spectra for water-soluble wood tar aerosol. **Table S1.** Chemical composition of the water-soluble wood tar material analyzed by the GC-MSD. **Figure S3.** Determination of particle mass concentration in mice exposure system. **Figure S4.** Mice inflammatory response after exposure to water soluble wood tar aerosol. **Figure S5.** Cytotoxicity of water-soluble wood tar extract. **Figure S6.** Mitochondria-response after exposure to water-soluble wood tar extracts. **Table S2.** List of Mus musculus and Homo sapiens primers.

## Data Availability

The datasets supporting the conclusions of this article are included within the article and its additional supplementary files.

## Abbreviations

BALF: Bronchoalveolar Lavage Fluid,
CYP2E: Cytochrome P450 2E
ECAR: Extracellular Acidification Rate
GPx-1: Glutathione Peroxidase-1
HO-1: Heme Oxygenase-1
IL: Interleukin
MDA: Malondialdehyde
MT-2: Metallothionein-2
MtDNAcn: Mitochondrial DNA Copy Number
NF-kB: Nuclear Factor Kappa B
OA: Organic Aerosol
OCR: Oxygen Consumption Rate
PAHs: Polyaromatic Hydrocarbons
PM: Particulate Matter
ROS: Reactive Oxygen Species
SMPS: Scanning Mobility Particle Sizer
TEM: Transmission Electron Microscopy
TNF-α: Tumor Necrosis Factor α
WSPs: Wood Smoke Particles
