# Supplementary material for: Mechanisms of lung toxicity induced by biomass burning aerosols
Source: Part Fibre Toxicol. 2020 Jan 20;17:4. doi: 10.1186/s12989-020-0337-x (PMC6971884; doi:10.1186/s12989-020-0337-x)
Supplement: Supplementary file 1 — Additional file 1. Figure S1. HR-Tof-AMS spectra for water-soluble wood tar aerosol. Figure S2. FT-IR spectra for water-soluble wood tar aerosol. Table S1. Chemical composition of the water-soluble wood tar material analyzed by the GC-MSD. Figure S3. Determination of particle mass concentration in mice exposure system. Figure S4. Mice inflammatory response after exposure to water soluble wood tar aerosol. Figure S5. Cytotoxicity of water-soluble wood tar extract. Figure S6. Mitochondria-response after exposure to water-soluble wood tar extracts. Table S2. List of Mus musculus and Homo sapiens primers. [file 12989_2020_337_MOESM1_ESM.docx]

**Mechanisms of lung toxicity induced by biomass burning aerosols**

Michal Pardo ^1*^, Chunlin Li ^1^, Quanfu He ^1^, Smadar Levin-Zaidman ^2^, Michael Tsoory ^3^, Qingqing Yu ^4,5^, Xinming Wang ^4,5,6^, Yinon Rudich ^1^

^1^ Department of Earth and Planetary Sciences, Weizmann Institute of Science, Rehovot 76100, Israel

^2^ Electron Microscopy Unit, Weizmann Institute of Science, Rehovot 76100, Israel

^3^ Department of Veterinary Resources, Weizmann Institute of Science, Rehovot, 761001, Israel

^4^ State Key Laboratory of Organic Geochemistry and Guangdong Key Laboratory of Environmental Protection and Resources Utilization, Guangzhou Institute of Geochemistry, Chinese Academy of Sciences, Guangzhou 510640, China

^5^ University of Chinese Academy of Sciences, Beijing 100049, China

^6^ Center for Excellence in Regional Atmospheric Environment, Institute of Urban Environment, Chinese Academy of Sciences, Xiamen 361021, China

* Corresponding Author:
Michal Pardo, Department of Earth and Planetary Sciences, Weizmann Institute of Science, Rehovot, 76100, Israel. Phone: +972 8 9344235; Fax +972-8-934-4124; Email: [Michal.levin@weizmann.ac.il](mailto:Michal.levin@weizmann.ac.il)

### Additional file 1

**Results**

***Water-soluble wood tar extract characterization***

Water soluble wood tar extract were characterized using various methodology: HR-Tof-AMS spectra in Figure S1 illustrates bulk chemical characterizations of water-soluble wood tar aerosol, four types of fragment ions were classified according to their elemental compositions as hydrocarbon fragment (C_x_H_y_^+^), oxygenated hydrocarbon fragments with one or more oxygen additions (C_x_H_y_O^+^ and C_x_H_y_O_z_^+^), and nitrogen containing fragment (C_x_H_y_O_i_N_p_^+^). The major oxygenated fragments (over 58%) derived from the wood tar extracts indicates their high oxidation state, resembling the oxygenated organic aerosol (OOA). Wood tar aerosol spectra comprise considerable amount (~1.8%) of nitrogen containing ions, in consistence with previous work reporting abundant organic nitrogen compounds (e.g., amides, nitro species, and organonitrates) in smoke particles [1, 2, 3]. Some typical fragments in the AMS spectra correspond to specific functional groups and chemical compounds help to extrapolate detailed chemical properties of the detected particles. The significant C_2_H_4_O_2_^+^ and C_3_H_5_O_2_^+^ ions trace the presence of levoglucosan or analog anhydrous sugars, which are commonly used as chemical markers for biomass burning emissions [4]. Typical hydrocarbon ions, e.g., C_2_H_2_^+^, C_3_H_3_^+^, C_6_H_6_^+^, and C_7_H_7_^+^, etc., denote the aromatic compositions in wood tar aerosol [5]. C_8_H_9_O_2_^+^ is a fragment from ionization of methoxy-phenols [5]. Moreover, C_2_H_3_O^+^ and CO_2_^+^ are critical carbonyl and carboxyl/peroxide functional markers, especially CO_2_^+^, higher intensity of CO_2_^+^ indicates higher oxidation degree of organic aerosol, thereby, results in higher CCN activity and volatility [4]. Above all, the water-soluble wood tar extracts consist of a matrix of anhydro sugars, aromatic, phenolic, and many other compounds carrying extensive carbonyl, carboxyl/ peroxide functional groups from AMS analysis. The tar aerosols have O/C and H/C molar ratios of 0.443 and 1.708, similar with values reported for ambient tar aerosol and related fire smoke carbonaceous emissions [5, 6].

The FT-IR spectra complement the chemical information inferred from HR-Tof-AMS measurement. In Figure S2, in addition to the pronounced -OH stretching of phenol and/or alcohol hydroxy groups in the 3550-3050 cm^-1^ region, the strong C=O vibration of carbonyl group at 1800-1650 cm^-1^ are seen. The strong C=O and -OH vibration together with C-O stretch (1100-1000 cm^-1^) suggest abundant acids, alcohols, ethers, or/and esters in the water-soluble tar aerosol, which is also supported by the AMS analysis in Figure S1. The aromatic C=C (1600, 1515 cm^-1^), the strong absorptions of typical sp^2^-aromatic C-H (~3060 cm^-1^), aliphatic C-H (3050-2800 cm^-1^), and unsaturated hydroxyl C=C-OH vibration (1050 cm^-1^) imply the contribution of alkyl substituted phenolic compounds in the particle [7, 8]. The FT-IR spectra acquired in this study present striking similarities with that of biomass burning related substances. In particular, the IR spectra in this study overlap burning related humic-like substance and tar balls in large scale [6, 9].

GC-MS results (Table S) in line with the AMS and FT-IR analysis give a clear insight into the molecular compositions in the tar aerosol. The GC-MS detected a large portion of sugars (e.g. levoglucosan, galactosan, manosan) in the sample which agrees well with the AMS measurements. Besides sugars, organic acids (such as resin acids, fatty acids and aromatic acids) and *n*-alkanes which presents in biomass burning aerosols [10, 11] are also observed in our sample. For most commonly detected PAHs in atmospheric particles, only a few of them (Acenaphthylene, Acenaphthene, Fluorine, Phenanthrene, Anthracene) are observed at trace levels (below 0.0002% in mass) while those highly carcinogenic species (e.g. Benzo[a,e]pyrene, Dibenzo[a,h]anthrancene, Benz[a]anthracene, Benzo[b,k,j]fluoranthene) are not detected at all (Table S2). Those identified molecules represent high proportions of ambient and laboratory smoldering emissions, suggesting that the water-soluble tar extracts generated in this study can be an effective proxy of filed fire tar aerosols, at least, the major fractions of water soluble matrix in ambient tar aerosols can be assessed in their toxicity using our tar aerosol.


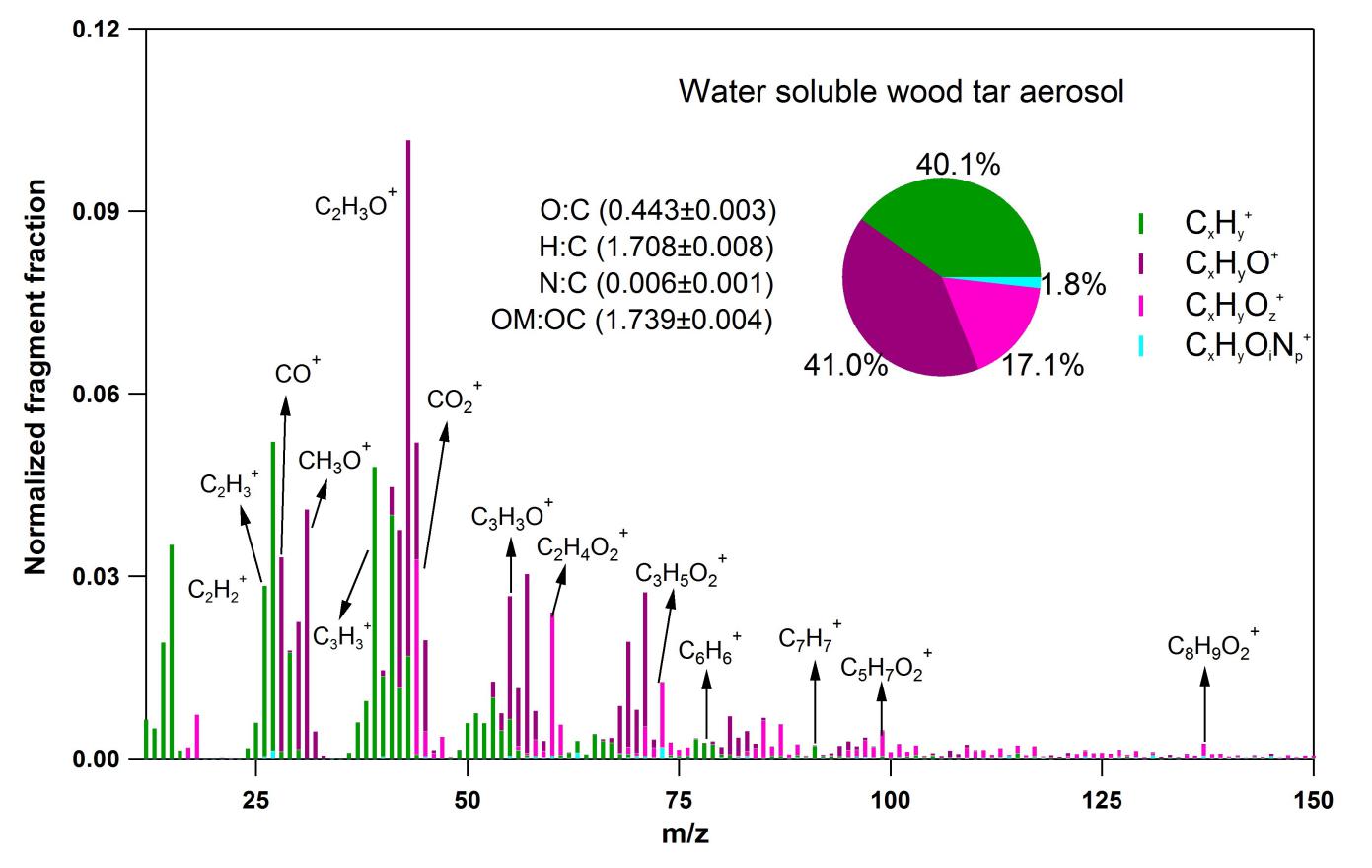
**Figure S1. HR-Tof-AMS spectra for water-soluble wood tar aerosol.** Signal for the fragment was normalized, and four chemical groups were classified based on their elemental compositions and marked in different color (x≥1, y≥1, z>1, i≥0, and p≥1), their contributions were pie-chart displayed.


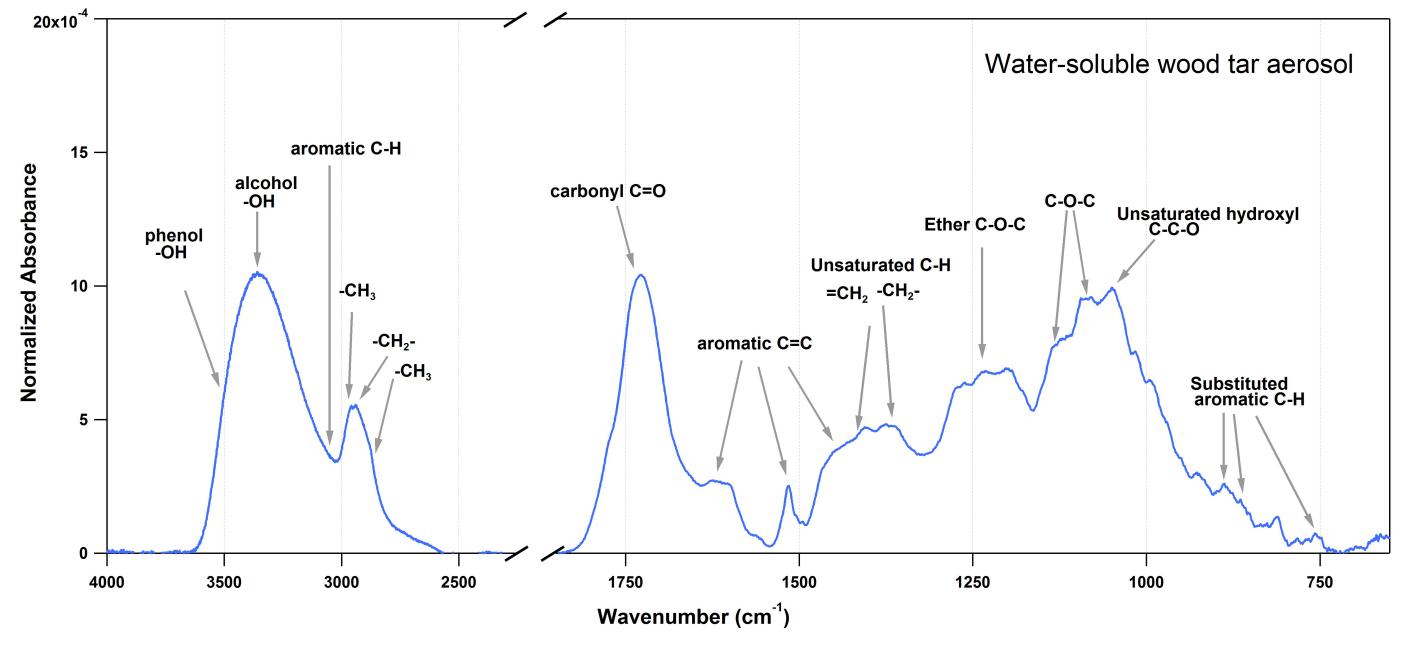


**Figure S2. FT-IR spectra for water-soluble wood tar aerosol**. The signal for the functional groups was normalized to the whole integrated spectra and assigned according to related work on biomass burning particles.

Table S1. Chemical composition of the water-soluble wood tar material analyzed by the GC-MSD.

| Compounds | Fraction (‱) |  | Compounds | Fraction (‱) |  | Compounds | Fraction (‱) |  | Compounds | Fraction (‱) |  | Compounds | Fraction (‱) |
| --- | --- | --- | --- | --- | --- | --- | --- | --- | --- | --- | --- | --- | --- |
| ***Sugars*** | |  | ***PAHs*** | |  | ***Saturated Acids*** | |  | ***Amines*** | |  | ***n-Alkanes*** | |
| Levoglucosan | 9.971 |  | Nathphalene | nd |  | Nonanoic acid | 0.013 |  | Hexadecanamide | 0.003 |  | Undecane | 4.893 |
| arabitol | 0.024 |  | Acenaphthylene | 0.014 |  | Decanoic acid | 0.004 |  | 9-Octdecenamide | 0.004 |  | Dodecane | 0.582 |
| mannitol | 0.002 |  | Acenaphthene | 0.019 |  | Undecanoic acid | 0.007 |  | Octadecanamide | 0.001 |  | Tridecane | 0.035 |
| inositol | 0.001 |  | Fluorine | 0.013 |  | Dodecanoic acid | 0.009 |  |  |  |  | Tetradecane | 0.009 |
| galactosan | 3.145 |  | Phenanthrene | 0.003 |  | Tridecanoic acid | 0.002 |  | ***Aromatic acids*** | |  | Pentadecane | 0.082 |
| mannosan | 4.924 |  | Anthracene | 0.001 |  | Tetradecanoic acid | 0.005 |  | Benzoic acid | 0.173 |  | Hexadecane | 0.042 |
|  |  |  | Fluoranthene | nd |  | Pentadecanoic acid | 0.002 |  | m-Toluic acid | 0.031 |  | Heptadecane | 0.019 |
| ***Dicarboxylic acid*** | |  | Acephenanthrylene | nd |  | Hexadecanoic acid | 0.131 |  | p-Toluic acid | 0.014 |  | Octadecane | 0.036 |
| Butanedioic Acid | 1.85 |  | Pyrene | nd |  | Heptadecanoic acid | 0.002 |  | 1,2-Phthalic acid | 0.141 |  | Nonadecane | 0.057 |
| Pentanedioic Acid | 1.068 |  | Retene | nd |  | Octadecanoic acid | 0.061 |  | 1,4-Phthalic acid | 0.027 |  | Eicosane | 0.029 |
| Hexanedioic Acid | 0.075 |  | Benzo(ghi)fluoranthene | nd |  | Nonadecanoic acid | 0.001 |  | 1,3-Phthalic acid | 0.033 |  | Heneicosane | 0.028 |
| Heptanedioic Acid | 0.208 |  | Cyclopenta(cd)pyrene | nd |  | Eicosanoic acid | 0.003 |  | 2-Hydroxybenzonic acid | 0.031 |  | Docosane | 0.02 |
| Octanedioic Acid | 0.575 |  | Benz(a)anthracene | nd |  | Heneicosanoic acid | 0.001 |  | 3-Hydroxybenzonic acid | 0.828 |  | Tricosane | 0.016 |
| Nonanedioic Acid | 1.31 |  | Chrysene/Triphenylene | nd |  | Docosanoic acid | 0.002 |  | 4-Hydroxybenzonic acid | 0.046 |  | Tetracosane | 0.013 |
| Decanedioic acid | 0.202 |  | Benzo(b)fluoranthene | nd |  | Tricosanoic acid | nd |  | 3,4-Dihydroxybenzoic acid | 0.001 |  | Pentacosane | nd |
|  |  |  | Benzo(k)fluoranthene | nd |  | Tetracosanoic acid | nd |  | Syringic acid | nd |  | Hexacosane | nd |
| ***Others*** | |  | Benzo(j)fluoranthene | nd |  | Pentacosanoic acid | nd |  | ferulic acid | nd |  | Heptacosane | nd |
| glycerol | 0.082 |  | Benzo(e)pyrene | nd |  | Hexacosanoic acid | nd |  | vanillic acid | 1.121 |  | Octacosane | nd |
| Erythritol | 0.034 |  | Benzo(a)pyrene | nd |  | Heptacosanoic acid | nd |  | 4-Methoxybenzoic acid | 0.08 |  | Nonacosane | nd |
| Dehydroabietic acid | 0.023 |  | Perylene | nd |  | Octacosanoic acid | nd |  | 3,4-Dimethoxy benzoic acid | 0.201 |  | Triacontane | nd |
| Abietic acid | 0.002 |  | Indeno(cd)fluoranthene | nd |  | Nonacosanoic acid | nd |  | 4-methyl-1,2-phthalic acid | 0.04 |  | Hentriacontane | nd |
| 7-Oxodehydroabietic acid | 0.015 |  | Indeno(cd)pyrene | nd |  | Triacontanoic acid | nd |  | 1,2,4-Benzenetricarboxylic | 0.015 |  | Dotriacontane | nd |
| Benz(de)anthracen-7-one | nd |  | Dibenzo[a,h]anthrancene | nd |  | Henriacontanoic acid | nd |  | 1,3,5-Benzenetricarboxylic | 0.005 |  | Tritriacontane | nd |
| malic acid | 0.643 |  | Picene | nd |  | Dotriacontanoic acid | nd |  | 1,2,4,5-Benzenetetracarboxy | 0.018 |  | Tetracontane | nd |
| glyceric acid | 0.021 |  | Benzo(ghi)perylene | nd |  |  |  |  |  |  |  | Pentatriacontane | nd |
| tartaric acid | 0.008 |  | Coronene | nd |  | ***Unsaturated acids*** | |  |  |  |  | Hexatriacontane | nd |
| citric acid | 0.054 |  |  |  |  | 9-Hexadecenoic acid | 0.017 |  |  |  |  |  |  |
|  |  |  |  |  |  | 9,12-Octadecanedienoic acid | 0.002 |  |  |  |  |  |  |

### nd means not detected

### *Exposure Assessment*

### Aerosols produced via nebulization of water-soluble extract from wood tar solution. These aerosols were used in mice exposure and were tested for particle size distribution, to calculate the particle mass exposure of the mice. A scanning mobility particle sizer (TSI, SMPS) and an additional condensation particle counter (TSI, CPC) were used to calculate the particle mass concentration in the aerosol flow. Specifically, the SMPS and CPC were connected to the exposure device via one of the six exposure tubes for mice maintenance. The other five were kept close as during real exposure. The pump side of the exposure device was blocked and a 15 ml tube was used as a trap for condensed liquid. While producing aerosols with the nebulizer, the flow from the exposure device was dried (RH<20%) by two diffusion driers (filled with silica gel) and introduced into the CPC and SMPS for total particle number concentration and size distribution measurement (Figure S3A). Meanwhile, a mass flow controller in parallel with the CPC and SMPS was installed for simulating the same total flow rate as for mice exposure (780 mL/min). SMPS and CPC data was collected for 30 minutes. The average size distribution data was obtained (Figure S3B) and the total particle number concentration of the SMPS was corrected by the data from CPC. Water extracts with tar material concentration of 1.43, 2.86, and 5.74 g/L were used for the size distribution measurement. Before changing solutions, the nebulizer was filled with pure water to clean the system until the measured particle number concentration is below 0.01% of the averaged concentration of the previous measurement. The total particle mass concentration in the flow from the exposure device is calculated based on the size distribution data using particle effective density of 1.35 g/cm^3^ [12]. Based on these measurement, the relationship between water-soluble extract concentration ([Tar extract]) and particle mass concentration (m_par_) in the flow was obtained by a power law fit (Figure S3C).

In order to evaluate exposure mass, a calculation using the minute volume was performed; the respiratory minute volume is the [volume](http://en.wikipedia.org/wiki/Volume) of gas [inhaled](http://en.wikipedia.org/wiki/Inhalation) or [exhaled](http://en.wikipedia.org/wiki/Exhalation) from a person's [lungs](http://en.wikipedia.org/wiki/Lung) per minute. The minute volume of mice is about 0.028 L/min or 0.04 m^3^ per day [13]. Based on the particle mass concentration (M_par_ =5593.9 + 5771.1 × [Tar extract]^2.4447), minute volume and exposure time, the mass of particles of an exposure to every individual mouse corresponds to 16 µg (for the 2 mg/ml initial concentration) and about 677 µg for the 10 mg/ml initial concentration.


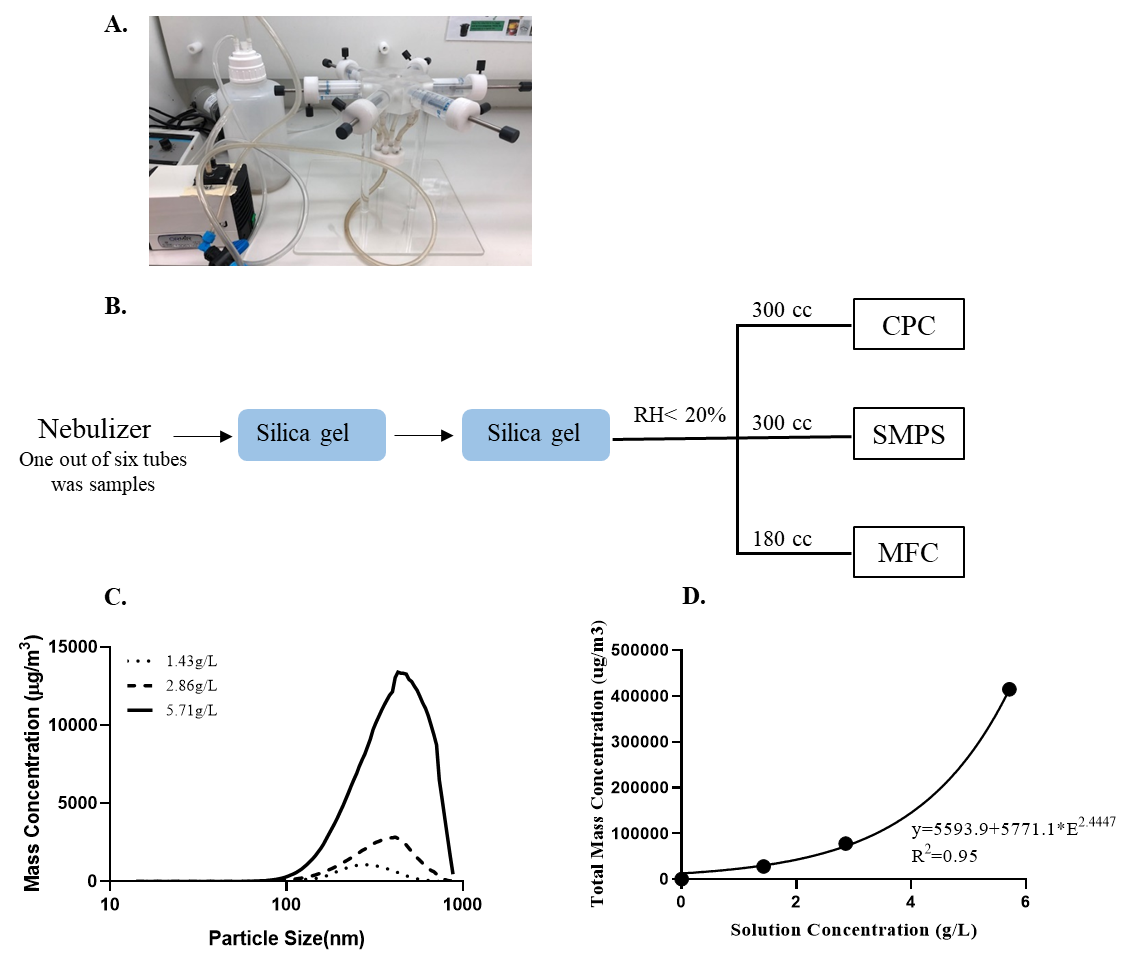


**Figure S3.** **Determination of particle mass concentration in mice exposure system**. (A) A photo of the exposure system, (B) Schematic diagram of the particle mass concentration measurement system. The wood tar aerosols are produced by a nebulizer and dried by silica gels. The flow was then introduced into a condensation particle counter (CPC, TSI) and a scanning mobility particle sizer (SMPS, TSI), (C) Particle mass distribution of wood tar aerosols, (D) Relationship between water extract solution concentration and the particle mass concentration in the flow.


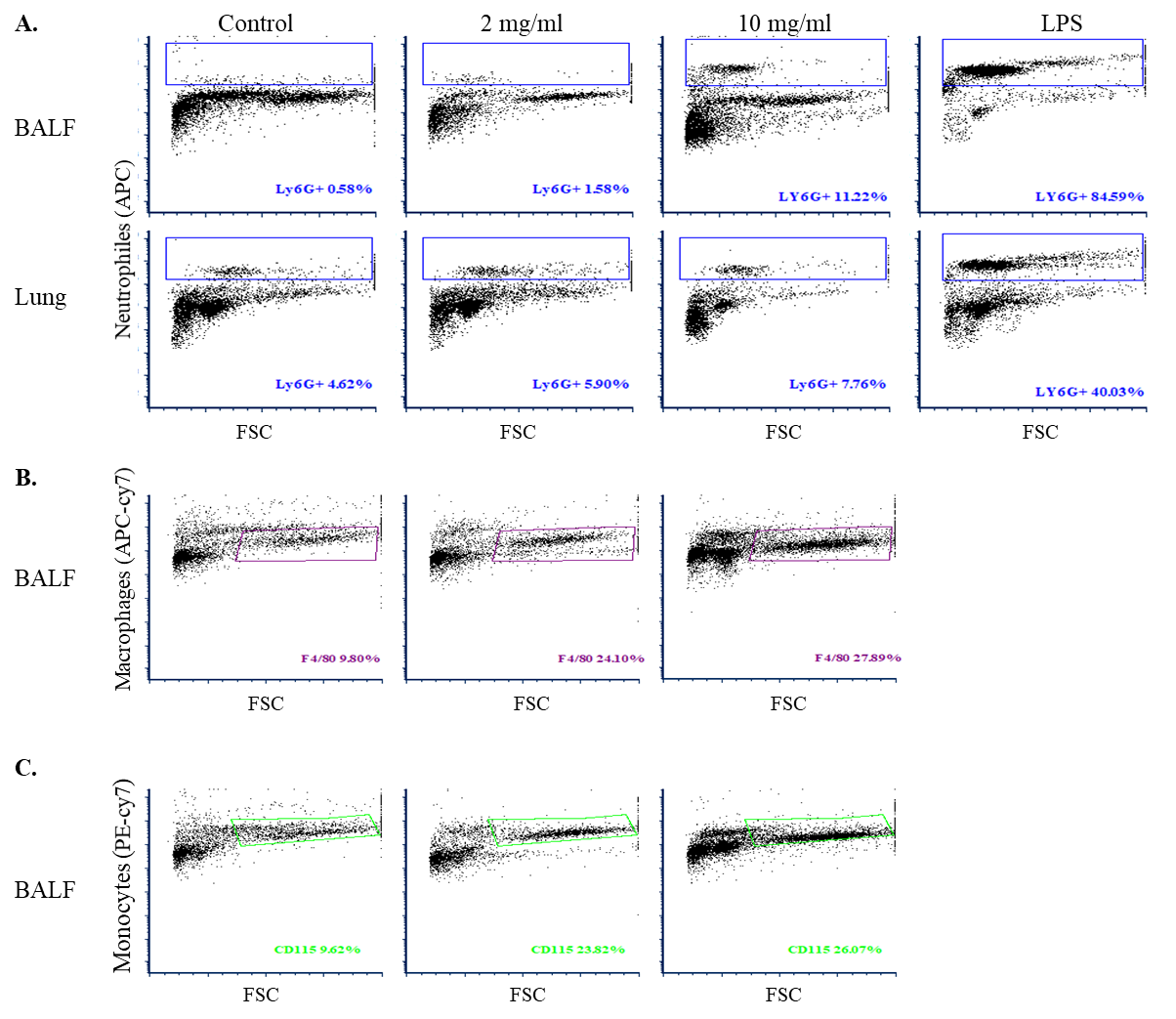


**Figure S4.** **Mice inflammatory response after exposure to water soluble wood tar aerosol**. Mice were exposed to aerosols generated by a nebulizer from the water soluble extracts from wood tar using individual single exposure model. Each exposure contained initial wood tar solution at concentration of 2 mg/ml or 10 mg/ml divided to the six mice for each concentration (*n*=6). Lipopolysaccharide (LPS) was used as positive control (*n*=4). The induction of inflammatory response was evaluated by flow cytometry with antibodies against different immune populations with different markers. (A) Histograms of neutrophils markers from BALF and lung tissue, the blue box indicates the gating by which the population is defined, (B) Histograms of macrophages markers from BALF, the purple box indicates the gating by which the population is defined, (C) histograms of monocytes markers from BALF, the green box indicates the gating by which the population is defined.

**Figure S5. *In vivo* transcription of real time PCR genes.** Quantitative analysis of targeted genes was performed by RT and real-time PCR for mRNA levels of (A) IL1β (B) TNF-α (C) IL-6 (D) HO-1 (E) MT-2 (F) CYP2E (G) Nrf2 (H) catalase and (I) GPx-1. Values are expressed as fold change of gene expression compared to a calibrator (endogenous control, β-Actin, HPRT). Data represent means ± SE; *n* = 6 mice per group. The means marked with different letters are significantly different from each other at p < 0.05.

**Figure S6. *In vitro* transcription of real time PCR genes.** Quantitative analysis of targeted genes was performed by RT and real-time PCR for mRNA levels of (A) IL1β (B) TNF-α (C) IL-6 (D) IL-8 (E) Nrf2 (F) catalase (G) Gpx-1 (H) Sod1 (I) Sod2 (J) Bax (K) caspase 3 and (L) bcl2. Values are expressed as fold change of gene expression compared to a calibrator (endogenous control, β-Actin, HPRT). Data represent means ± SD; The data are expressed as the mean±SD. Means marked with different letters are significantly different from each other at p < 0.05.


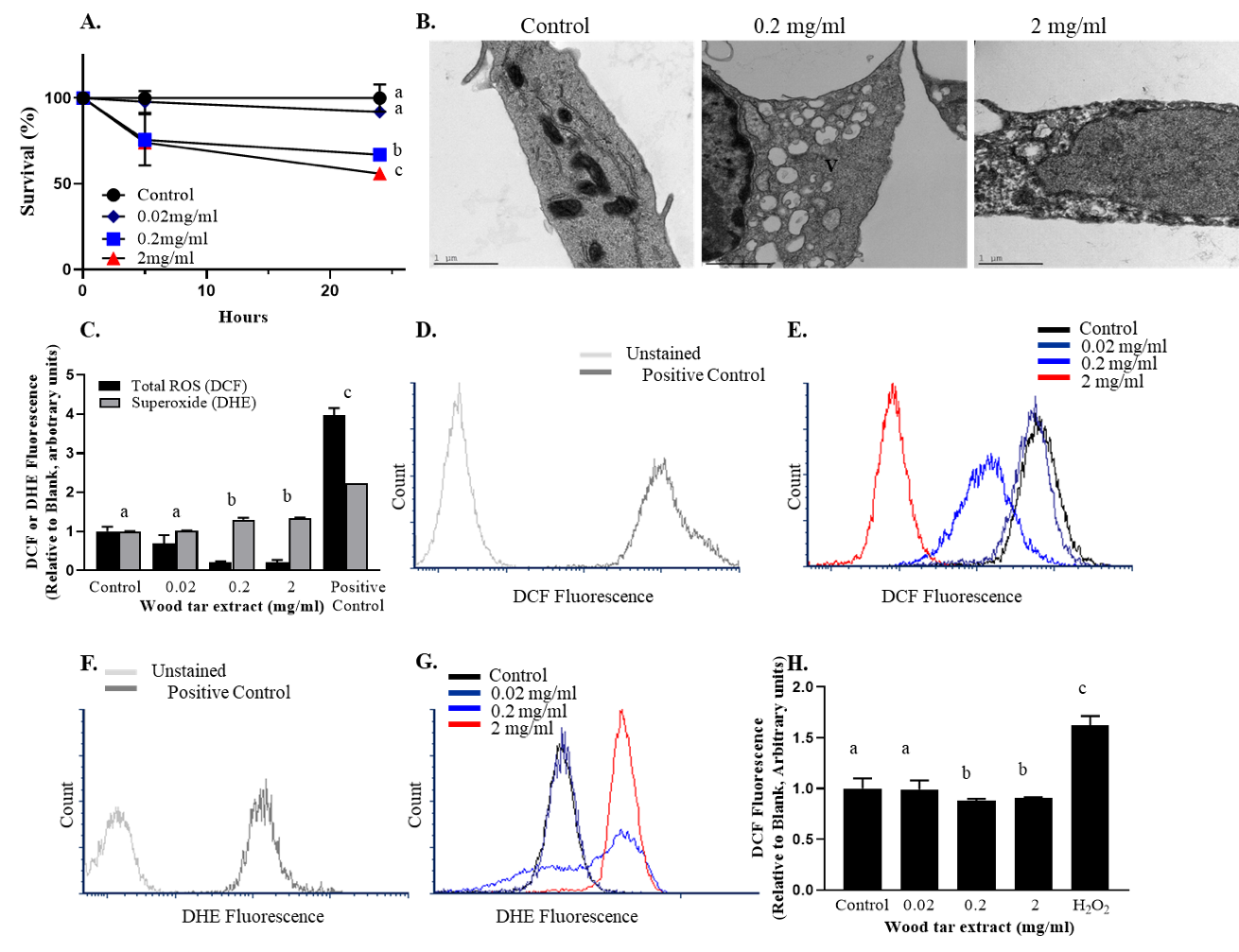


**Figure S7.** **Cytotoxicity of water-soluble wood tar extract**. Water-soluble wood tar extracts were used in 0.02 mg/ml, 0.2 mg/ml and 2 mg/ml concentrations, 5 or 24 h prior to analysis as specified. (A) WST-1 assay, (B) TEM images of the cells 24 hours after exposure of control- blank treated cells, wood tar solution 0.2 mg/ml treated cells, and 2 mg/ml -treated cells. MIT, mitochondria; N, nucleus; V, vacuoles, (C) Intracellular ROS and superoxide anion were measured using H_2_DCF-DA and DHE stains respectively, detection was made by flow cytometry (ZE5 Cell Analyzer, Bio-Rad), (D) flow cytometry histogram for DCF fluorescence with unstained cells and hydrogen peroxide (H_2_O_2_, 50 µM treated cells-positive control), (E) flow cytometry histogram for DCF fluorescence of cells treated with wood tar solutions, (F) flow cytometry histogram for DHE fluorescence with unstained cells and “antimycin A” treated cells-positive control, (G) flow cytometry histogram for DHE fluorescence of cells treated with wood tar solution, (H) intracellular ROS were measured using H_2_DCF-DA and the detection was made by micro plate reader (VT 05404, Bio-Tech Instruments) at 488/532 nm. The means marked with different letters are significantly different from each other at p < 0.05.


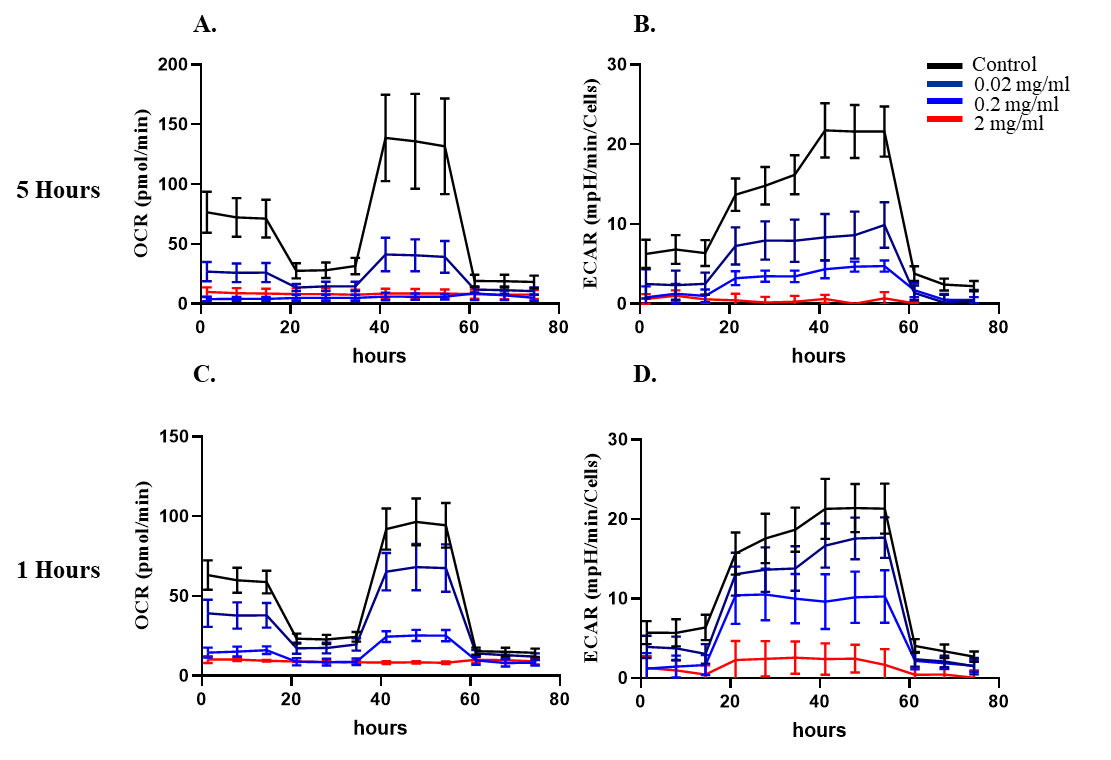


**Figure S8.** **Mitochondria-response after exposure to water-soluble wood tar extracts**. Water-soluble wood tar extracts were used in 0.02 mg/ml 0.2 mg/ml and 2 mg/ml concentrations, 1 or 5 h prior to analysis as specified. Determination of the mitochondrial oxygen consumption rate (OCR, mitochondrial respiration) and extracellular acidification rate (ECAR, glycolysis) were performed as indicated in the methods according to the manufacturer's instructions (Agilent). Following 5 hours exposure, (A) selected experiment showing basal and OCR mean following injections of the inhibitors and substances. And (B) selected experiments showing basal and ECAR mean following injections of the inhibitors and substances. Following 1 hour of exposure (C) OCR and (D) ECAR. These experiments were performed in triplicate and were repeated twice.

Table S2. List of Mus musculus and Homo sapiens primers

| *Mus musculus* | Genes | Forward '5-3' | Revers '5-3' |
| --- | --- | --- | --- |
| interleukin 1 beta | Il1β | GGATGATGATGATAACCTGC | CATGGAGAATATCACTTGTTGG |
| Tumor necrosis factor | Tnf-α | GGTTATCTTGCTAGGTCTTTG | GATCCCTACAAATGATGGAG |
| interleukin 6 | Il6 | AAGAAATGATGGATGCTACC | GAGTTTCTGTATCTCTCTGAAG |
| heme oxygenase 1 | OH-1 | CATGAAGAACTTTCAGAAGGG | TAGATATGGTACAAGGAAGCC |
| Metallothionein 2 | MT-2 | TGTACTTCCTGCAAGAAAAG | GGCTTCTACATGGTCTATTTAC |
| cytochrome P450, family 2, subfamily e, polypeptide 1 | CYP2E | AGGCTGAGACAGGGATACCA | CGTCCACTTCAGAGCTTCCT |
| nuclear factor, erythroid derived 2, like 2(Nfe2l2) | Nrf2 | TCTCCTCGCTGGAAAAAGAA | AATGTGCTGGCTGTGCTTTA |
| catalase | catalase | CTCCATCAGGTTTCTTTCTTC | CAACAGGCAAGTTTTTGATG |
| glutathione peroxidase 1 | gpx-1 | TGGCTTGGTCATTCTGGGC | CCCACCTGGTCGAACATACTT |
| glutathione reductase | Gsr | GTTCACACAGGTTAAGGAAG | TATTCAGATTCAGGCCCTTAG |
| β-actin, mus musculus | β-actin | GATGTATGAAGGCTTTGGTC | TGTGCACTTTTATTGGTCTC |
| *Homo sapiens* Genes |  |  |  |
| interleukin 1 beta | Il1β | TCC CAG CCC TTT TGT TGA | TTA GAA CCA AAT GTG GCC GTG |
| Tumor necrosis factor | Tnf-α | GAA CAT CCA ACC TTC CCA AA | GGT TGA GGG TGT CTG AAG GA |
| interleukin 6 | Il6 | GGC ACT GGC AGA AAA CAA CC | GCA AGT CTC CTC ATT GAA TCC |
| interleukin 8 | Il8 | TCT GGC AAC CCT AGT CTG CT | AAA CCA AGG CAC AGT GGA AC |
| heme oxygenase 1 | OH-1 | GAGAAAGCAAGTGGCTCACC | TGACGGACCTGGTTCTTACC |
| nuclear factor, erythroid derived 2, like 2(Nfe2l2) | Nrf2 | CAACCCTTGTCACCATCTCA | GTGTTCTCACATTGGGCATC |
| catalase | catalase | TTCTTGGATGCAAAGTGCTG | GTCAGCTGAACCCGATTCTC |
| glutathione peroxidase 1 | gpx-1 | CTGGTCCTGTTGATCCCAGT | CTGACACCCGGCACTTTATT |
| bcl-2-like protein 4 | Bax | CCAAGAAGCTGAGCGAGTGT | ATCCTCTGCAGCTCCATGTT |
| BCL2 apoptosis regulato | bcl2 | GTTCAAACAAGACGCCAACA | ATGGAAGGCCACATCTGAAC |
| Caspase 3 | caspase 3 | TGGAATTGATGCGTGATGTT | TGGCTCAGAAGCACACAAAC |
| Superoxide dismutase 1 | SOD1 | CACTCTAAGAAACATGGTGG | GATCACACGATCTTCAATGG |
| Superoxide dismutase 2 | SOD2 | AAGTTCAATGGTGGTGGTCA | GTTAGGGCTGAGGTTTGTCC |
| β-actin | β-actin | TCGTGCGTGACATTAAGGAG | CCATCTCTTGCTCGAAGTCC |
| Hypoxanthine Phosphoribosyltransferase 1 | HPRT | TGTGGTATGGTATGGCTTGC | GGTGAAAGAGCAGGTGAACA |
| tRNA LEU (UUR) | tRNA LEU (UUR) | CACCCAAGAACAGGGTTTGT | TGGCCATGGGTATGTTGTTA |
| β2-Microglobulin | β2-Microglobulin | TGCTGTCTCCATGTTTGATGTATCT | TCTCTGCTCCCCACCTCTAAGT |

**Methods**

***Water-soluble wood tar extract chemical analysis***

Chemical analysis, including bulk chemical determination, functional group characterization and molecular identification was conducted to the water-soluble wood tar aerosol using multiple MS techniques and FT-IR system. A high-resolution time-of-flight aerosol mass spectrometer (HR-Tof-AMS, Aerodyne) was applied to measure the bulk composition of non-refractory organic matter in particles, alternating V and W modes were used to scan the organic fragments in m/z range of 12~350. In total, four ion groups were classified based on elemental compositions of these fragments from V-mode measurements, as C_x_H_y_^+^, C_x_H_y_O^+^, C_x_H_y_O_z_^+^ (H_x_O_y_^+^ included), and C_x_H_y_O_i_N_p_^+^, respectively, where x≥1, y≥1, z≥2, p≥1, and i≥0. Elemental ratios of O/C and H/C were estimated from integration of all fragments measured via W-mode. Detailed information refers to [14]. The particles nebulized from the water-soluble extracts of wood tar were dehydrated through multiple silica gel denuders and impacted onto KBr plate. Functional groups in the deposited samples were measured using FT-IR (Thermo Scientific Nicolet^TM^ 6700). FT-IR operated in the wavelength range of 4000-400 cm^-1^ at resolution of 2 cm^-1^, totally 32 scans were averaged. The final FT-IR spectra was subtracted from blank KBr baseline. For accurate quantative chemical analysis, 191.2 mg dried water-soluble wood tar material was analyzed by an Agilent gas-chromatography coupled with a mass spectrometer detector (GC-MSD). Briefly, the material was dissolved in 4 mL methanol by sonification. Lab-prepared internal standard solution (containing isotopic labeled n-alkanes, acids, PAHs, sugars etc.) and one quarter of the solution were re-dissolved in the mixed solvent dichloride methane (DCM)/methanol (1:1, vol/vol). Then the solution was filtered, dried, and concentrated to ~2mL. The residual was separated into two parts. One was blown to dryness and derivatize carboxylic acids to methyl esters. The methylated extract was analyzed for PAHs, alkanes, fatty acids and other nonpolar compounds. The other portion was blown to dryness for silylation and was analyzed for compounds containing hydroxyl groups and other polar functional groups such as sugars and aromatic acids. Details of the derivation method and operation of the GC-MSD can be found in previous publications [15, 16]

**References**

1. Laskin A, Smith JS, Laskin J. Molecular Characterization of Nitrogen-Containing Organic Compounds in Biomass Burning Aerosols Using High-Resolution Mass Spectrometry. Environmental Science & Technology. 2009;43 10:3764-71; doi: 10.1021/es803456n. <https://doi.org/10.1021/es803456n>.

2. Lin P, Bluvshtein N, Rudich Y, Nizkorodov SA, Laskin J, Laskin A. Molecular Chemistry of Atmospheric Brown Carbon Inferred from a Nationwide Biomass Burning Event. Environmental Science & Technology. 2017;51 20:11561-70; doi: 10.1021/acs.est.7b02276. <https://doi.org/10.1021/acs.est.7b02276>.

3. Xie M, Chen X, Hays MD, Holder AL. Composition and light absorption of N-containing aromatic compounds in organic aerosols from laboratory biomass burning. Atmos Chem Phys. 2019;19 5:2899-915; doi: 10.5194/acp-19-2899-2019. <https://www.atmos-chem-phys.net/19/2899/2019/>.

4. Ng NL, Canagaratna MR, Zhang Q, Jimenez JL, Tian J, Ulbrich IM, et al. Organic aerosol components observed in Northern Hemispheric datasets from Aerosol Mass Spectrometry. Atmos Chem Phys. 2010;10 10:4625-41; doi: 10.5194/acp-10-4625-2010. <https://www.atmos-chem-phys.net/10/4625/2010/>.

5. He LY, Lin Y, Huang XF, Guo S, Xue L, Su Q, et al. Characterization of high-resolution aerosol mass spectra of primary organic aerosol emissions from Chinese cooking and biomass burning. Atmos Chem Phys. 2010;10 23:11535-43; doi: 10.5194/acp-10-11535-2010. <https://www.atmos-chem-phys.net/10/11535/2010/>.

6. Tóth Á, Hoffer A, Pósfai M, Ajtai T, Kónya Z, Blazsó M, et al. Chemical characterization of laboratory-generated tar ball particles. Atmos Chem Phys. 2018;18 14:10407-18; doi: 10.5194/acp-18-10407-2018. <https://www.atmos-chem-phys.net/18/10407/2018/>.

7. Cain JP, Gassman PL, Wang H, Laskin A. Micro-FTIR study of soot chemical composition—evidence of aliphatic hydrocarbons on nascent soot surfaces. Physical Chemistry Chemical Physics. 2010;12 20:5206-18; doi: 10.1039/B924344E. <http://dx.doi.org/10.1039/B924344E>.

8. Bruns EA, Perraud V, Zelenyuk A, Ezell MJ, Johnson SN, Yu Y, et al. Comparison of FTIR and Particle Mass Spectrometry for the Measurement of Particulate Organic Nitrates. Environmental Science & Technology. 2010;44 3:1056-61; doi: 10.1021/es9029864. <https://doi.org/10.1021/es9029864>.

9. Zhong M, Jang M. Dynamic light absorption of biomass-burning organic carbon photochemically aged under natural sunlight. Atmos Chem Phys. 2014;14 3:1517-25; doi: 10.5194/acp-14-1517-2014. <https://www.atmos-chem-phys.net/14/1517/2014/>.

10. Simoneit BRT, Elias VO. Organic tracers from biomass burning in atmospheric particulate matter over the ocean. Marine Chemistry. 2000;69 3:301-12; doi: <https://doi.org/10.1016/S0304-4203(00)00008-6>. <http://www.sciencedirect.com/science/article/pii/S0304420300000086>.

11. Zhang YX, Shao M, Zhang YH, Zeng LM, He LY, Zhu B, et al. Source profiles of particulate organic matters emitted from cereal straw burnings. J Environ Sci (China). 2007;19 2:167-75. <https://www.ncbi.nlm.nih.gov/pubmed/17915724>.

12. Li C, He Q, Schade J, Passig J, Zimmermann R, Meidan D, et al. Dynamic changes in optical and chemical properties of tar ball aerosols by atmospheric photochemical aging. Atmos Chem Phys. 2019;19 1:139-63; doi: 10.5194/acp-19-139-2019. <https://www.atmos-chem-phys.net/19/139/2019/>.

13. W. Bide R, J. Armour S, Yee E. Estimation of Human Toxicity From Animal Inhalation Toxicity Data: 1. Minute Volume-Body Weight Relationships Between Animals And Man. 1997.

14. DeCarlo PF, Kimmel JR, Trimborn A, Northway MJ, Jayne JT, Aiken AC, et al. Field-Deployable, High-Resolution, Time-of-Flight Aerosol Mass Spectrometer. Analytical Chemistry. 2006;78 24:8281-9; doi: 10.1021/ac061249n. <https://doi.org/10.1021/ac061249n>.

15. Ding X, Wang X-M, Gao B, Fu X-X, He Q-F, Zhao X-Y, et al. Tracer-based estimation of secondary organic carbon in the Pearl River Delta, south China. Journal of Geophysical Research: Atmospheres. 2012;117 D5; doi: 10.1029/2011jd016596. <https://agupubs.onlinelibrary.wiley.com/doi/abs/10.1029/2011JD016596>.

16. Ding X, Wang X-M, Zheng M. The influence of temperature and aerosol acidity on biogenic secondary organic aerosol tracers: Observations at a rural site in the central Pearl River Delta region, South China. Atmospheric Environment. 2011;45 6:1303-11; doi: <https://doi.org/10.1016/j.atmosenv.2010.11.057>. <http://www.sciencedirect.com/science/article/pii/S1352231010010289>.
